# Evolutionary Stability Optimizer (ESO): A Novel Approach to Identify and Avoid Mutational Hotspots in DNA Sequences While Maintaining High Expression Levels

**DOI:** 10.1021/acssynbio.1c00426

**Published:** 2021-12-20

**Authors:** Itamar Menuhin-Gruman, Matan Arbel, Niv Amitay, Karin Sionov, Doron Naki, Itai Katzir, Omer Edgar, Shaked Bergman, Tamir Tuller

**Affiliations:** † School of Mathematical Sciences, The Raymond and Beverly Sackler Faculty of Exact Sciences, Tel Aviv University, Tel Aviv, Israel 6997801; ‡ Shmunis School of Biomedicine and Cancer Research, The George S. Wise Faculty of Life Sciences, Tel Aviv University, Tel Aviv, Israel 6997801; § School of Electrical Engineering, The Iby and Aladar Fleischman Faculty of Engineering, Tel Aviv University, Tel Aviv, Israel 6997801; ∥ Department of Biomedical Engineering, Tel Aviv University, Tel Aviv, Israel 6997801; ⊥ School of Medicine, The Sackler Faculty of Medicine, Tel Aviv University, Tel Aviv, Israel 6997801; # The Sagol School of Neuroscience, Tel Aviv University, Tel Aviv, Israel 6997801

**Keywords:** genetic stability, computer-aided design (CAD), evolutionary stability optimizer (ESO), mutational hotspots, epigenetic hotspots, stability and expression trade-off

## Abstract

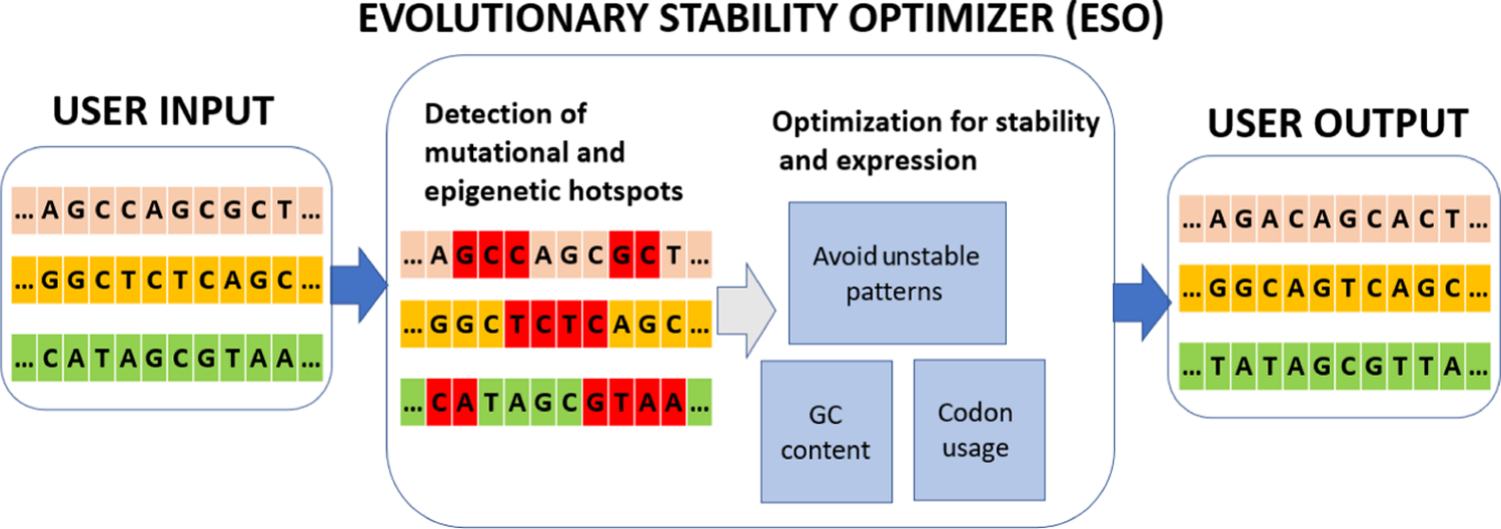

Modern synthetic biology procedures rely on the ability to generate stable genetic constructs that keep their functionality over long periods of time. However, maintenance of these constructs requires energy from the cell and thus reduces the host’s fitness. Natural selection results in loss-of-functionality mutations that negate the expression of the construct in the population. Current approaches for the prevention of this phenomenon focus on either small-scale, manual design of evolutionary stable constructs or the detection of mutational sites with unstable tendencies. We designed the Evolutionary Stability Optimizer (ESO), a software tool that enables the large-scale automatic design of evolutionarily stable constructs with respect to both mutational and epigenetic hotspots and allows users to define custom hotspots to avoid. Furthermore, our tool takes the expression of the input constructs into account by considering the guanine-cytosine (GC) content and codon usage of the host organism, balancing the trade-off between stability and gene expression, allowing to increase evolutionary stability while maintaining the high expression. In this study, we present the many features of the ESO and show that it accurately predicts the evolutionary stability of endogenous genes. The ESO was created as an easy-to-use, flexible platform based on the notion that directed genetic stability research will continue to evolve and revolutionize current applications of synthetic biology. The ESO is available at the following link: https://www.cs.tau.ac.il/~tamirtul/ESO/.

## Introduction

**Figure 1:**
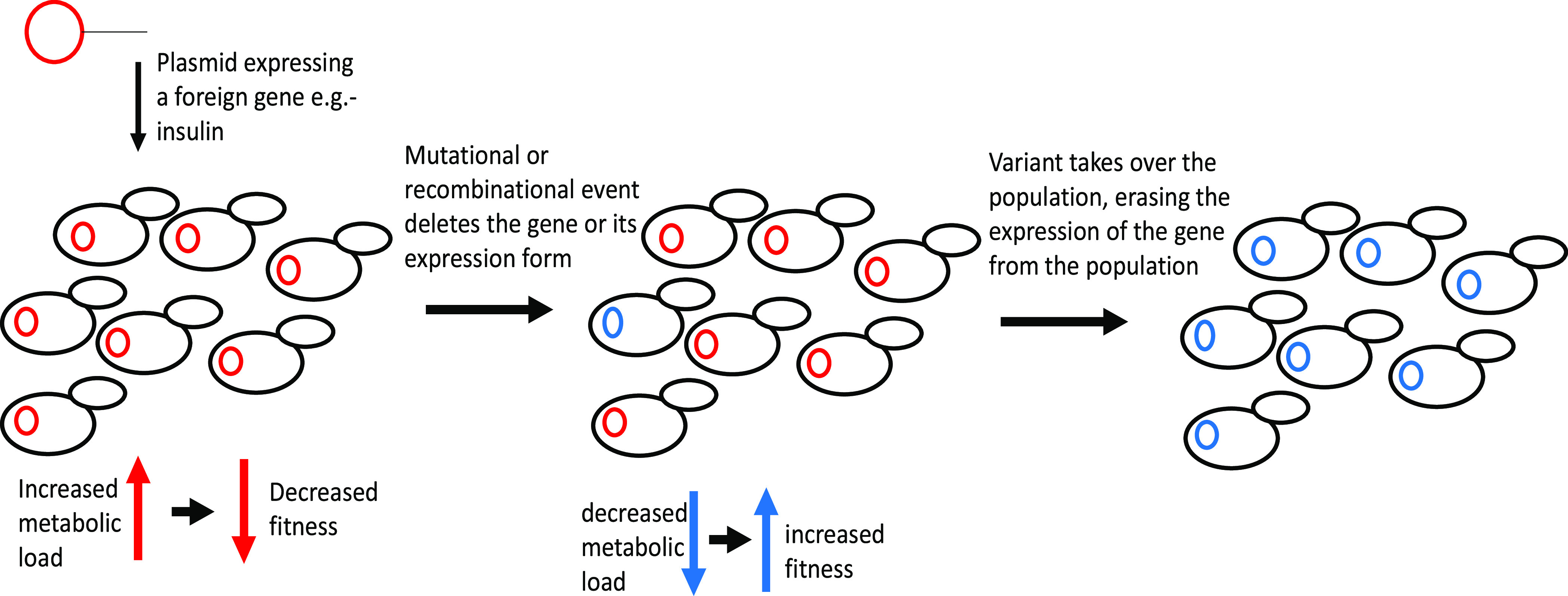
Selection process of modified populations. Illustration of the selection process to the most-fit variant in a population of genetically modified microorganisms, resulting in their evolutionary instability.

Recent advances in the quickly evolving field of synthetic biology have led to the development of various genetic circuits for therapeutics and bioproduction applications.^1−5^ However, once such a construct is inserted into a host organism, it imposes an additional burden on the host because of (a) the metabolic load of synthesizing unnecessary RNAs and proteins and (b) heterologous genetic parts that interfere with native cellular processes.^6^ Both phenomena significantly reduce host fitness, leading to the presence of strong selective pressure against the exogenous genetic circuit.^7,8^ Therefore, loss-of-function mutations that damage the construct are likely to be selected for, diminishing or negating altogether the activity of the circuit.^6,9^ Because of their increased fitness, the mutated individuals will eventually take over the population (Figure 1). These mutations could render synthetic-biology-related products obsolete and require constant maintenance. Moreover, circuits with high evolutionary stability are known to have low expression levels.^10^ Thus, designing a DNA sequence to specifically withstand evolutionary failure while preserving or increasing expression levels is an important goal for synthetic biology.

**Figure 2:**
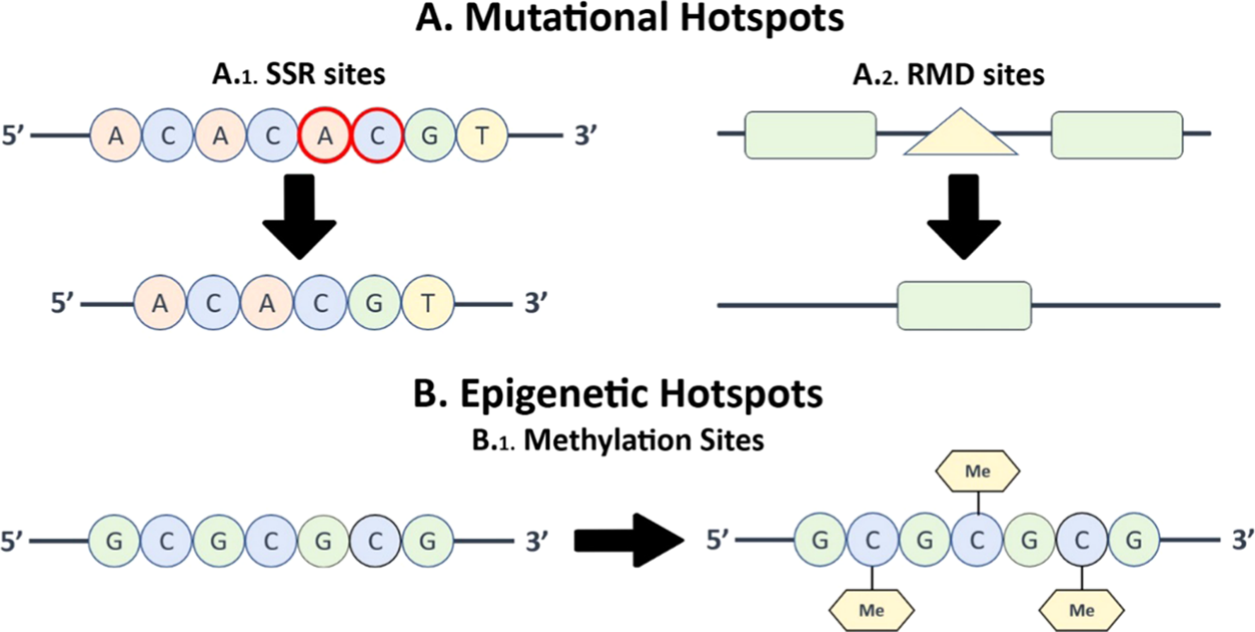
Hotspots detected by the Evolutionary Stability Optimizer (ESO). (A) Mutational hotspots. Simple Sequence Repeats (SSRs) are repeating short sequences to which, due to polymerase slippage mistakes, a short sequence can be added or deleted. Repeat-Mediated Deletions (RMDs) stem from long sequences appearing in different parts of the gene, in which a misread causes the deletion of an intermediate sequence. (B) Epigenetic hotspots: methylation sites; the attachment to methyl groups can cause a change in the DNA’s folding, potentially leading to lesser or no gene expression.

Generally, a small number of mutational hotspots in a given construct are responsible for most of the mutations accumulated in the construct^11,12^ (Figure 2). Their presence can destabilize any genetic circuit in nearly any organism. Two major examples for these hotspots are (a) Simple Sequence Repeats (SSRs), sequences rich with repeating short motifs that increase the chance for inaccurate replication,^13^ and (b) Repeat-Mediated Deletions (RMDs), deletion events arising from unwanted recombination between long repeated sequences.^14^ Another type of genetic instability that can affect a construct is an epigenetic change in the expression patterns of the genes involved.^15^ Specifically, the addition of a methyl group to adenine- or cytosine-containing sites is known to repress the inserted genes in insectoid and mammalian host cells.^15−19^

These instability hotspots, if detected in advance, can be manually removed when planning a synthetic construct. However, the field of generic tools for the improvement of mutational stability is surprisingly neglected. One of the most common web tools assisting in such an analysis is the Evolutionary Failure Mode (EFM) calculator,^20^ which enables the prediction of potential mutational vulnerabilities in a given DNA sequence. Using empirical data collected from various studies,^21−25^ the calculator predicts the probability of mutation in the hypermutable sites (sites with a high probability of mutation) of SSR and RMD, and compares them with the Base Pair Substitution (BPS) rate (see the Methods section for details). High-scoring sites within the genetic sequence are far more likely to be mutated and, subsequently, erased or modified to significantly increase the evolutionary stability. While the EFM tool predicts SSR and RMD sites, it does not provide a way to delete them from the designed genetic sequence.

Another recent tool, the Nonrepetitive Parts Calculator (NRPC),^26^ is based on machine learning and graph algorithms presented in ref (27). In this work, given a maximal allowed length of repeating sequences between different genetic parts, thousands of such parts are generated and analyzed. This allows for a straightforward design of synthetic sequences while avoiding RMD sites and significantly reducing the likelihood of mutation.

However, these tools are unsuited for the current direction of genetic work, which becomes more systematic and large-scale, since they both perform single sequence analysis. Furthermore, neither of the tools addresses all mutation types listed above: the EFM calculator does not predict areas of epigenetic instability, and the NRPC only considers RMD sites. Most importantly, their design principles do not consider the required trade-off between contradictory demands of evolutionary stability and high expression levels. Our tool considers this trade-off and increases evolutionary stability while aiming at maintaining expression levels rather than decreasing expression.

In this paper, we introduce the next generation of the EFM calculator, called the evolutionary stability optimizer (ESO): a robust tool for automatic optimization of large-scale sequences for optimal genetic and epigenetic stability. This tool provides an end-to-end solution for the design of stable constructs: it enables a large-scale detection of SSR, RMD, methylation, and custom sites in multiple sequences at once and offers optimization of these sequences with respect to genetic stability while maintaining expression levels.

## Results and Discussion

### ESO Features

The EFM calculator developed by Jack et al.^20^ is a highly useful, computationally efficient web tool. The calculator finds and ranks SSR and RMD sites within a user’s input sequence, allowing the users to manually delete or modify these sites as needed.

Desiring to create a more intuitive, flexible tool, which enables both DNA engineering and gene expression improvement, we designed the ESO. Our hope is that it will provide a tool to generate stable, highly expressed genes to a larger userbase, with much lower costs in terms of time and effort. For this purpose, we included several important improvements on the detection mechanism provided by previous tools:

#### Large-Scale Analysis

**Figure 3:**
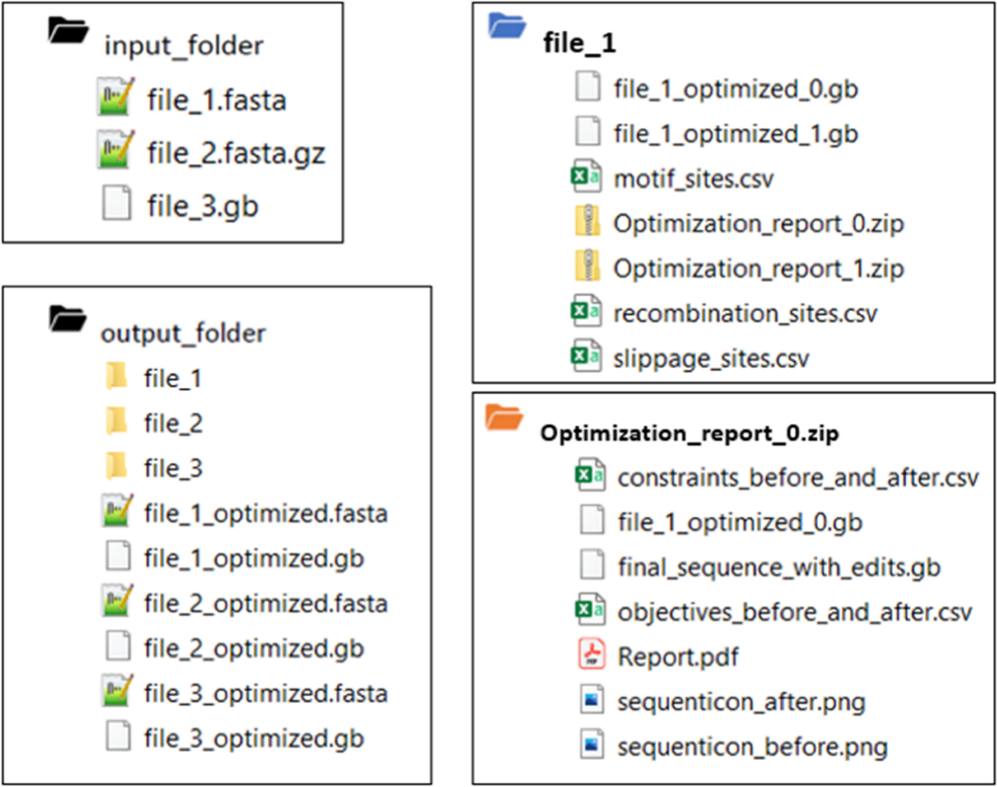
ESO’s input and output. The ESO receives an input folder that includes Fasta or Genbank files, either compressed or uncompressed (upper left block). Its output may include the corresponding optimized sequences in either the Fasta or Genbank format, as selected by the user. It also includes one folder for each input file (lower left block). Each output folder consists of CSV tables detailing the sites found, optimized subsequences in the Genbank format, and may also include an optimization report consisting of several files (upper right block). The optimization report includes the final sequence in the GenBank format, the sequenticon of the sequence before and after the changes, and the summary of the changes (lower right block).

The EFM calculator only enables the analysis of one sequence at a time, requiring manually inserting data and exporting results. For larger projects with many sequences, this would be a significant bottleneck, leading to a waste of time and possible file confusion. To address this issue in our software, the input is a directory, and all sequences in it are analyzed. The results are placed in an output directory, in a hierarchy-maintaining order, allowing the analysis of several sequences at once. Moreover, if the optimization option is being selected, a unique icon is provided to each sequence (a sequenticon; see https://github.com/Edinburgh-Genome-Foundry/sequenticon), allowing visual differentiation between sequences that otherwise might be confused with one another (Figure 3). Finally, for those not wishing to go into the analysis but rather desiring only the final results, one output file is provided per input file, with all optimized subsequences.

#### Consideration of Methylation Sites

As discussed above, mammalian and insectoid cells are much more sensitive to methylation sites than to SSRs and RMDs. Thus, to obtain optimal results for these cells, methylation must be considered. Using the methylation detection mechanism (see “Methods” for details), our software locates the sites most likely to match the existing known methylation sites.

#### Consideration of Alternative Sites

The search for methylation sites is based on Position-Specific Scoring Matrices (PSSMs) provided by Wang et. al.^28^ PSSMs are a commonly used tool in computational biology for the identification of motifs, in which the probability of each nucleotide in a subsequence is calculated in a position-dependent manner. Our software is designed to be modular, providing support for updated or different optimization requirements; users may provide their own PSSM matrices for sites to be avoided, providing greater customizability for unique engineering needs.

#### Automatic Optimization

The EFM calculator returns a list of hypermutable sites, with their location and ranking. This requires the user to spend significant time and effort manually correcting the sequence, often reaching suboptimal results. In our software, we designed an optimization engine that avoids the identified hotspots, does not change “locked” regions (i.e., regions designated by the user not to be changed), regulates the guanine-cytosine (GC) content, and increases the frequency of optimal codons. In addition to hotspot detection, the users are provided with a final, ready-to-use sequence, optimized for stability and expression. Thus, the ESO provides an end-to-end solution, a concept that is yet to exist in the field of genomic stability analysis.

For any given input sequence, the optimization procedure involves two steps: (a) optimized codon usage and required GC content; (b) avoid mutational patterns (SSR, RMD, and methylation or custom sites when relevant) detected by the previous module in the semioptimized sequence, while maintaining the codon usage bias and GC content as much as possible. This is done while avoiding changes to locked regions. This two-step strategy allows the algorithm to generate a sequence that is closer to optimum and only then deals with mutational hotspots. Thus, the probability that new problematic sites will appear after optimization decreases dramatically.

GC content optimization refers to the maintenance of the frequency of GC nucleotides within a specified range. The algorithm splits the sequence into windows of a specified size and optimizes within each window. The user may choose to regulate the GC content according to the principles suitable for the specific host. For instance, in *Saccharomyces cerevisiae*, the lower the GC content, the more stable is the sequence; it has been proved that genes with high GC had a substantially elevated rate of mutations—both single-base substitutions and deletions.^29^

In codon usage bias optimization, the algorithm replaces codons to match their frequency to the corresponding frequency in the host organism.^30^ The underlying assumption is that the genome of the host went through selective pressure for stability and expression in some form. Thus, by matching the sequence to the host, it will likely have higher levels of stability and expression as well. The optimization methods are “use best codon”, “match codon usage”, and “harmonize RCA”, all described in the DNA chisel paper.^31^

#### Synthetic Biology Case Study

**Figure 4:**
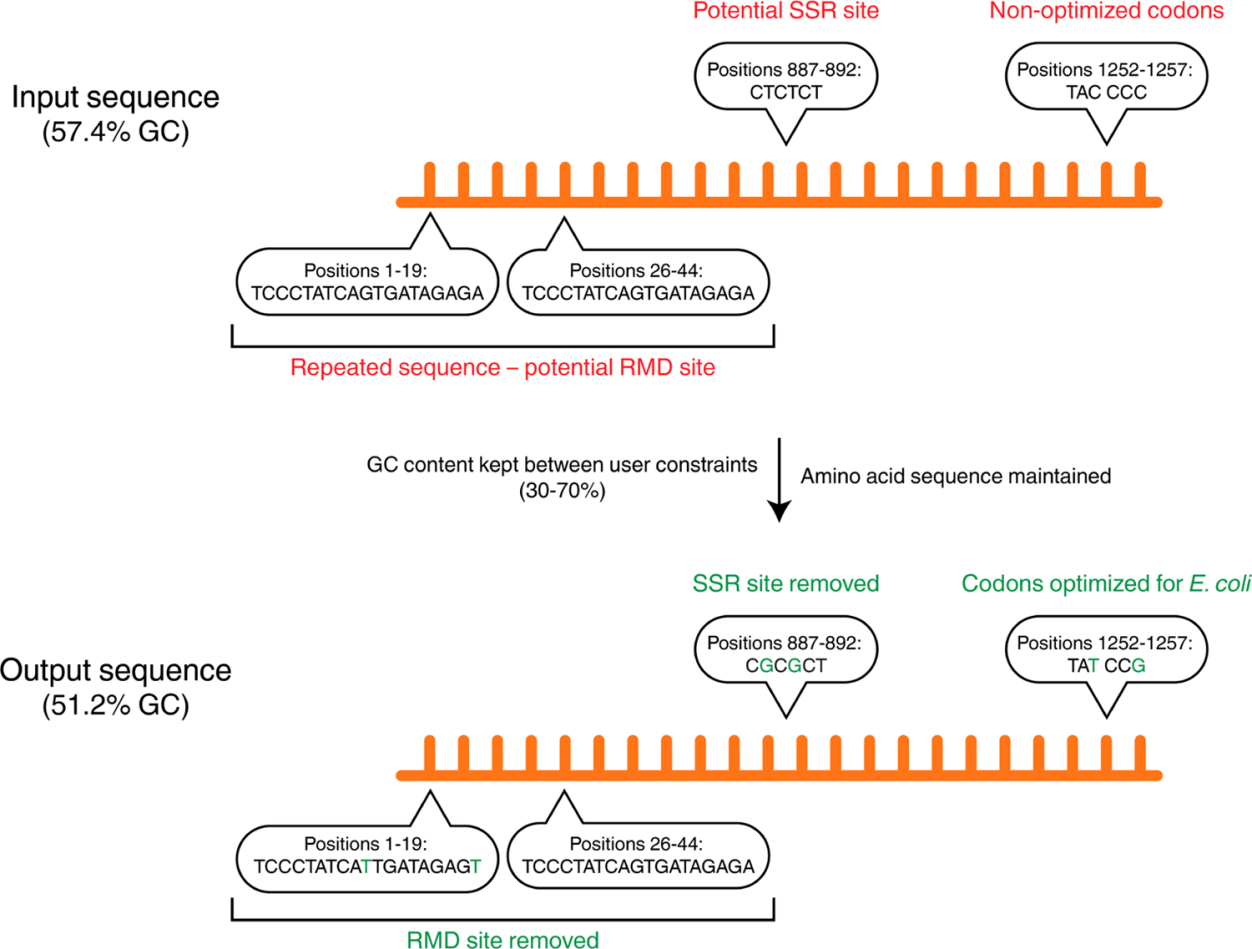
Case study. An illustration of the changes induced in the BBa_I13604 reporter construct, optimizing the gene for expression in *E. coli* and removing mutational and epigenetic hotspots.

As a case study, we used a reporter construct, BBa_I13604, from SynBioHub.^32^ The construct’s sequence was used as an input for the ESO, optimizing it for expression in *Escherichia coli* and constraining the GC content to be between 30 and 70% (Figure 4). All in all, 186 nucleotides were changed—optimizing the sequence’s codon usage and removing five potential recombination sites and 26 potential slippage sites while keeping the GC content within the constraints and preserving the amino acid sequence. The full input and output files can be found on our website (https://www.cs.tau.ac.il/~tamirtul/ESO/).

#### User Interface

**Figure 5:**
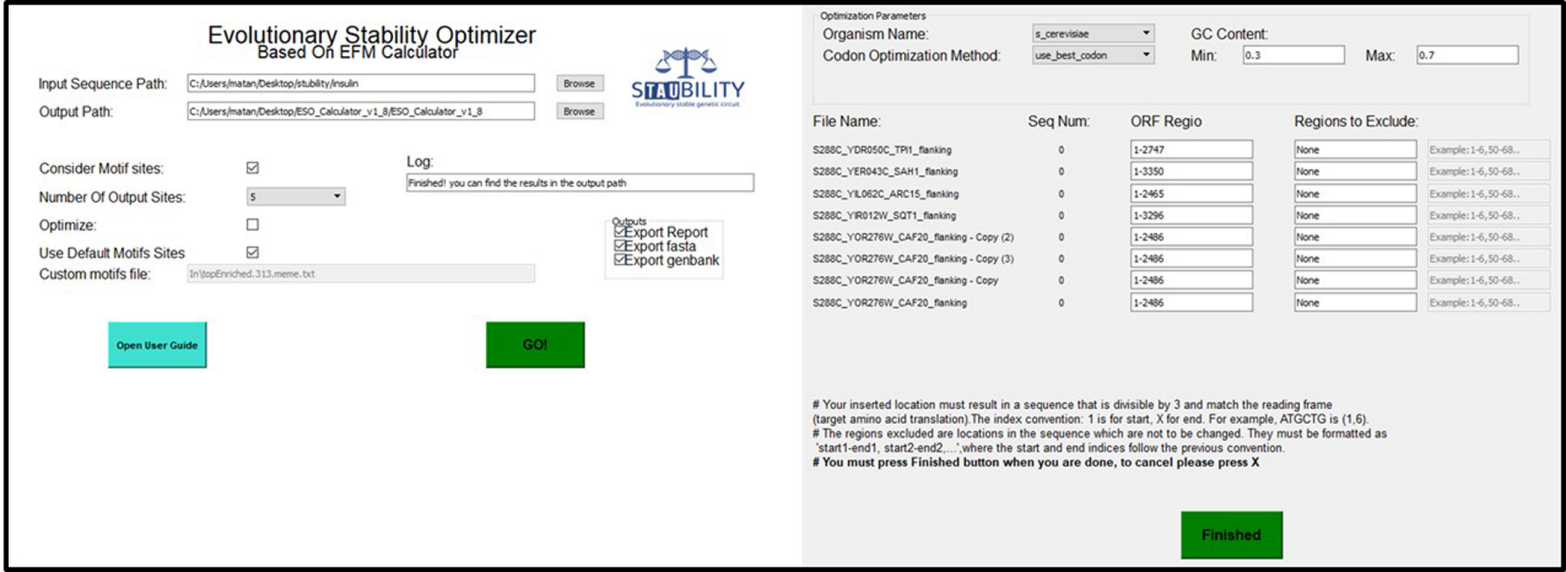
ESO’s GUI. The ESO main screen (left) and optimization screen (right). In the main screen, the user selects an input directory, whose sequences will be analyzed, and in the output directory, the results will be stored. In addition, the user can define whether to consider methylation motifs, custom motifs, or none, how many sites to consider, whether to design an optimized sequence or just return a list of sites to be corrected, in which format, and whether to include a full report. In the optimization screen, the user may define which organism and which method will be used to optimize codon usage, the bounds on GC content, ORF regions, and locked regions. If more than one sequence appears in a file, they will be given a running index called Seq Num. Note that the ORF length must be divisible by 3 for codon optimization.

To provide an end-to-end solution and enable the abovementioned analysis, we developed user-friendly software. We wrapped this software in a graphical user interface (GUI, Figure 5), downloadable as an application to the user’s computer, allowing greater computational capabilities.

### ESO Accurately Predicts the Evolutionary Stability of Endogenous Genes

**Figure 6:**
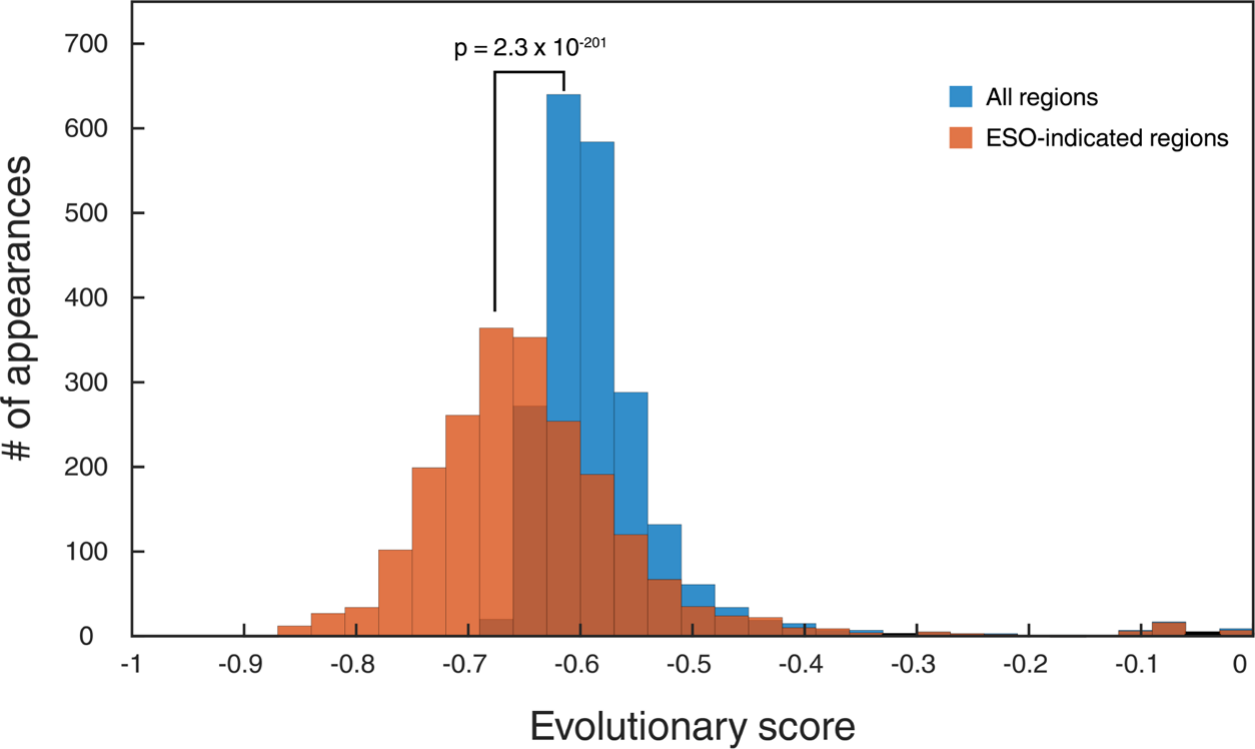
Rate4Site evolutionary scores. Evolutionary score histogram of ESO-indicated 5mers (red) and randomly selected 5mers (blue) and the number of appearances (*Y* axis). Higher evolutionary score signifying higher conservation (*X* axis). Significance level of difference was calculated using the Wilcoxon rank-sum test.

To demonstrate the efficiency and robustness of the ESO, we analyzed the evolutionary stability of residues marked as unstable by our software. We hypothesized that the areas marked by the ESO would have a lower conservation score than the average region, as they are genetically unstable. For this analysis, we used all 6008 *S. cerevisiae* genes from NCBI^33^ and employed our pipeline mirroring ConSurf^34^ (see the Methods section for details); briefly, each gene was aligned to its homologous sequences, found by BLAST,^35^ and the conservation of each position in the gene was calculated, based on that multiple sequence alignment (MSA), using Rate4Site.^36^ The final per-position evolutionary score is between −1 and 1, with a more positive score signifying higher conservation. We then analyzed the genes in our ESO program, which predicted evolutionarily unstable areas: for each region indicated by the ESO to be unstable, we calculated the average of the nucleotides’ five lowest conservation scores. To calculate a baseline for comparison, we randomly divided the entire gene into segments, each 5mers long (5 being the average unstable region length found by the ESO). Applying a scoring method similar to the one described above, we calculated the conservation score of all those individual segments and compared the randomly generated sites with those the ESO predicted to be unstable (Figure 6). Using the Wilcoxon rank-sum test, we found that the ESO-indicated regions were significantly less conserved than the randomly selected ones (*p* = 2.3 × 10^–201^).

To ensure the robustness of our approach, we repeated this method—this time taking the lowest three or four scores at each region instead of five; the conservation scores of random and ESO-indicated regions were significantly different as well (*p* = 1.05 × 10^–11^ and 7.7 × 10^–49^, respectively). We used the average of at least three lowest scores since a mutation mediated by SSR would cause a single nucleotide level event (deletion, insertion, or substitution), potentially rendering the region evolutionarily stable once more. Therefore, we do not predict that the whole area indicated by the ESO would be evolutionarily unstable, but rather that it will contain highly unstable residues. In addition, this approach reduces sensitivity to the wobble position.

This analysis demonstrates that the areas chosen and modified by the ESO are indeed expected to be less evolutionarily conserved; it also implies that the ESO software successfully predicts areas that are evolutionarily unstable and automatically offers a new, optimized sequence, which is expected to have enhanced evolutionary stability.

It has been previously shown that there is a trade-off between high expression levels and evolutionary stability.^11,12^ This is to be expected, as a large metabolic load of the construct leads to a larger difference in fitness between colonies that stopped expressing the construct to those still expressing it. However, this does not mean that increasing the evolutionary stability of a construct inherently decreases its expression levels. While it is possible to increase the evolutionary stability by decreasing the expression level, using the ESO, it is possible to increase the evolutionary stability while maintaining expression levels. A goal for future research will be to focus on this trade-off and find a method in which it is possible to simultaneously increase expression levels and evolutionary stability.

## Conclusions

As the field of synthetic biology keeps evolving, the need for generic tools enabling the design of stable genetic constructs increases rapidly.^37−42^ Our ESO software tool outperforms the existing tools in the field in several aspects. Combining mutational hotspots, such as RMD and SSR, with epigenetic hotspots prediction in one tool allows a better analysis of eukaryotic organisms. Alternatively, the tool enables avoidance of custom sites, providing a solution for custom engineering needs. It simplifies the large-scale analysis of multiple sequences. In addition, by applying automatic optimization for GC content and codon usage bias while avoiding mutational hotspots, the ESO provides output sequences optimized for stability, while maintaining expression levels. The solutions are presented in a simple and attractive user interface.

The benefits of using our software are reflected not only in saving time but also in lowering the costs of DNA design. Optimized sequences prevent human error and are more likely to succeed, reducing the likelihood that the process will need to be repeated. Using this software can aid individual researchers, as well as biotechnology companies, in developing new products.

Our ESO was designed using experiments and empirical data,^20−25,31^ giving further confidence that our computational design should work well experimentally. As the synthetic biology field is expanding, more research will be done on mutational rates in different organisms and other biological aspects that can affect mutations and their detection mechanism. Our modular design will incorporate this research, further improving future analysis.

Our tools with a guideline can be downloaded from https://www.cs.tau.ac.il/~tamirtul/ESO/.

## Methods

### Calculation of RMD and SSR Sites

The original EFM calculator^20^ considers three forms of mutation: SSR, RMD, and BPS, the latter giving a baseline mutational probability for comparison. From these, a Relative Instability Prediction (RIP) score is calculated as follows
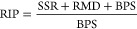
1This score can be thought of as “how much more likely is the sequence to mutate when also considering SSR and RMD sites versus considering only BPS errors?”. It gives a measure of how unstable a sequence is and receives its minimal value of 1 for the case of no SSR and no RMD mutational hotspots.

The following equations are based on empirical data collected from refs (21−25). The data were fitted with a log-linear approximation, providing generational mutation rates for *E. coli*. These rates are expected to be correlative with highly mutable sites in other organisms.

*SSRs* are sites composed of a repeating short sequence, causing potential polymerase slippage. For instance, the following sequence is an SSR: (AT)(AT)(AT)(AT); it has a base unit length (*L*) of 2 and number of units (*N*) of 4. The calculator considers SSR sites if they have (*N* ≥ 3, *L* ≥ 2), e.g., ATATAT, or (*N* ≥ 4, *L* = 1), e.g., AAAA. Denoting the generational mutation rate as μ, the SSR score of a site is calculated as follows
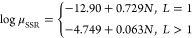
2

These rates are based on the empirical data collected by ref (20).

RMDs are long (*L* ≥ 16), identical sites appearing in different locations in the sequence, causing potential recombination faults. The recombination probability between two sites is based on their length *L* and the distance between them *L*_s_ and is calculated as follows
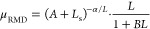
3where *A* = 5.8 ± 0.4, *B* = 1465.6 ± 50.0, and α = 29.0 ± 0.1 were found empirically.^21^

BPS is the probability of spontaneous mutations. It is empirically estimated in ref (25) as μ_BPS_ = 2.2 × 10^–10^ based on genome sequencing of *E. coli* mutation accumulation lines.

Note that all empirical findings were estimated for *E. coli*; although the probability of mutation will be different for other organisms, the ranking of hypermutable sites is approximately maintained.

### Calculation of Methylation Sites

As previously stated, the epigenetic inheritance process of methylation has a much more dominant effect on activation and expression in mammalian and insectoid cells (e.g., Chinese hamster ovary (CHO) cells). In the following analysis, we provide a method for the detection of highly probable methylation sites.

**Figure 7:**
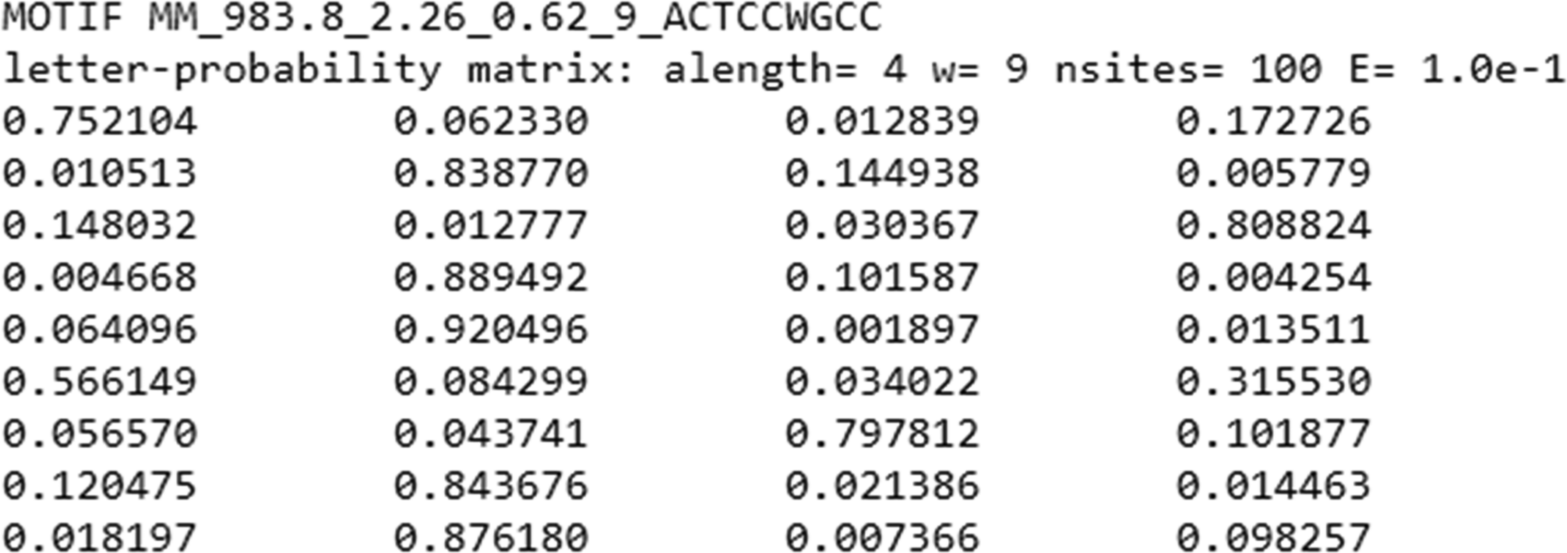
Example of a motif’s PSSM. The first line details the motif name. The second line details, in order, the alphabet length, the length of the motif, number of source sites, and E-value. The columns are ordered as ACGT and the rows by the nucleotide index in the motif. Each row gives a probability distribution for the appropriate index.

A motif is a sequence pattern that occurs repeatedly in a group of related sequences. The Multiple Expectation maximizations for Motif Elicitation (MEME) Suite is a collection of tools for the discovery and analysis of sequence motifs. Motifs are represented as position-dependent nucleotide probability matrices, describing the probability of each nucleotide per position in the pattern. In a study published by Wang et al.,^28^ 313 methylation motifs were identified and analyzed in brain, liver, and pancreatic cells. The reported motifs in Wang’s database (http://wanglab.ucsd.edu/star/MethylMotifs/) are presented in the MEME minimal format (Figure 7).

This database details, per methylation site, what is the likelihood of seeing a certain nucleotide sequence. This is commonly called a Position Probability Matrix (PPM). By normalizing each probability with the nucleotide background probability in the database’s host organism and taking the logarithm of these values, a Position-Specific Scoring Matrix (PSSM) is generated. This scoring mechanism is a common method in the field for scoring the likelihood of various sites.

For each genetic subsequence, the following calculation from Bayesian statistics provides us with the theoretical basis for estimating its probability of being a methylation site
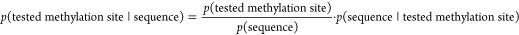
4

 is described by the PSSM score, and *p*(tested methylation site) is assumed to be uniform. Thus, the site and likelihood can be estimated by finding the site maximizing the PSSM score. The score indicates the probability of being a methylation site, and the higher the score, the more likely it is a methylation site. Thus, we can find the most likely sites within a sequence and rank them, finding the sites most in need of deletion or editing. This database is highly comprehensive and allows evaluating *p*(sequence|tested methylation site) with high accuracy. However, we note that it does not provide an estimate of the methylation process’s strength or the likelihood of methylation itself, given a methylation site.

It is important to mention that the user may choose to use alternative PSSM matrices, in the MEME minimal format as well. This standard format allows users to import custom motifs from alternative sources, allowing avoidance of sites dictated by individual engineering needs.

### Optimization Engine

The optimization process provided by the ESO utilizes the Python package DNA chisel, version 3.2.5,^31^ allowing for optimization of DNA sequences divisible by 3 with respect to a set of constraints and objectives. The following constraints are implemented: Enforce Translation (match the target amino acid translation for the ORF), Enforce GC content (in windows of 50 nucleotides), Match Pattern (for maintaining locked sites), and Avoid Pattern (for avoiding the mutational hotspots detected). The objective is Codon Optimization based on the usage table provided by the python-codon-tables package (https://pypi.org/project/python-codon-tables/) for the following organisms: *Bacillus subtilis*, *Caenorhabditis elegans*, *Drosophila melanogaster*, *E. coli*, *Gallus gallus*, *Homo sapiens*, *Mus musculus*, *Mus musculus domesticus*, and *S. cerevisiae*. For computational considerations, we offer avoidance of the 10 most probable sites from each type (SSR, RMD, and methylation or custom motif).

### Pseudo-code

#### Inputs

- Input folder path
- Output folder path
- Whether to compute motif scores
- PSSM file (PSSM of methylation sites supplied)
- Minimal and maximal allowed GC content
- Optimization method
- Organism
- ORF regions
- Regions not to be changed

#### Algorithm

1. Input
  : Read each Fasta or Genbank file in the input folder and divide into separate sequences.
2. First optimization:
  Define the optimization objective (‘use_best_codon’, ‘match_codon_usage’, ‘harmonize_rca’) and target organism. If no organism is specified, only the constraints will be resolved. Define constraints, minimal and maximal GC content in sequence, maintain codon translation in the ORF region, avoid changing nucleotides in locked regions. GC content will be enforced on each subsequence with length 50.
3. Recombination sites:
  Divide the sequence into subsequences of length16, find those appearing more than once, and merge together if they are subsequent to find longer sequences. Grade according to “RMD and SSR sites’ calculation” in the Methods section.
4. Polymerase slippage sites:
  Divide the sequence into subsequences of all lengths 1≤L≤15; for each subsequence, test if identical to the next. Filter and grade according to “RMD and SSR sites’ calculation” in the Methods section.
5. Motif sites
  : Scan the sequence and its reverse-complement using PSSM matrices and find maximal PSSM score per index. Keep it only if the PSSM score is larger than 0 (greater than the score for a random sequence with background frequencies). PSSM matrices for methylation are provided with the software and custom sites may be used.
6. Second optimization: Define the optimization objective and constraints similar to the first optimization, with added constraints:

- change each subsequence of length 15 in the first site of each recombination pair (ensuring that shorter recombination sites do not remain)
- change sequence in the motif location
- change alternating repeating units in slippage sites

7. Output: Zip with the final sequence as GenBank file, optimization report, and sequenticon for ease of use. In addition, output csv summarizing recombination, polymerase slippage, and motif sites. In addition to zip, output the final sequence separately, as well as Genbank and Fasta files of the final sequences joined together, matching input files, for ease of use.

### Conservation Score Analysis

For each of the 6008 *S. cerevisiae* genes, we utilized a pipeline mirroring that of the ConSurf program.^34^ We could not use the ConSurf website directly since it does not provide a way to run large-scale calculations on more than one gene at a time.

All genes were run through BLAST^35^ to find similar sequences in the NR database (excluding *S. cerevisiae* sequences); genes with less than 20 BLAST hits were discarded since this indicates that these genes are not conserved. For each gene, we then filtered highly similar BLAST hits using CD-HIT^43^ and created multiple sequence alignments (MSAs) of each gene with its BLAST hits using MAFFT.^44^ These MSAs were used as an input for the Rate4Site program, which lies at the heart of ConSurf. The per-base evolutionary conservation scores calculated by Rate4Site^36^ are the basis of the conservation analysis described here and in the Results and Discussion section. All in all, 2136 genes were used in the final analysis.

**Figure 8:**
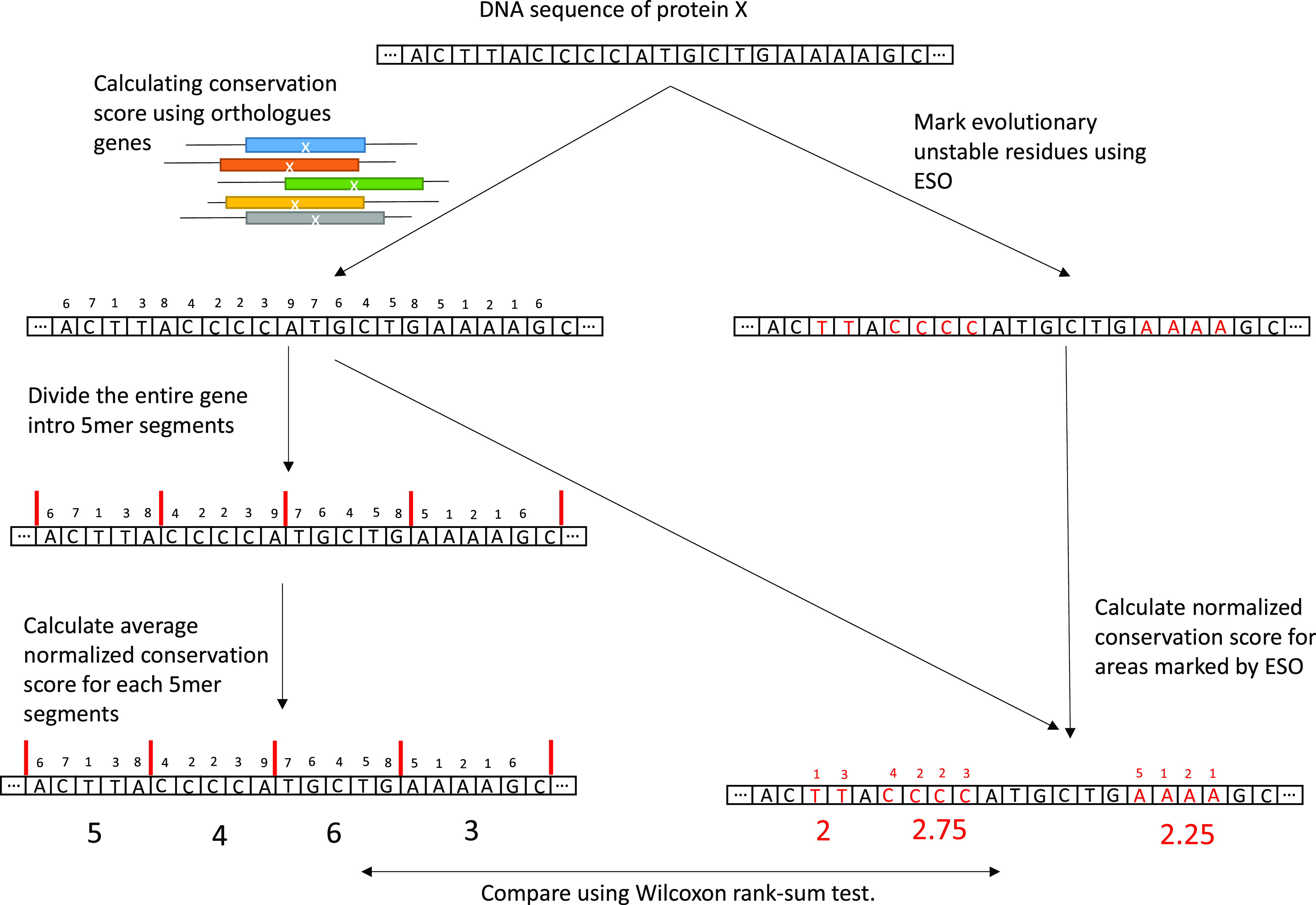
Conversation score analysis scheme. 2136 *S. cerevisiae* conserved genes were analyzed; for each gene, a per-nucleotide conversation score was calculated using Rate4Site. Utilizing the ESO, evolutionarily unstable areas were marked (red *numbers and letters*). The median of the average conversation score of the lowest 3–5 nucleotides in each randomized section/ESO site was then calculated for the entire protein.

Using this tool, the average conservation score of each protein was calculated and compared with the mean conservation score of regions indicated by the ESO to be evolutionarily unstable in the same protein (Figure 8).
